# Microbial β‐lactone natural products: An annotated selection of World Wide Web sites relevant to the topics in microbial biotechnology

**DOI:** 10.1111/1751-7915.12600

**Published:** 2016-12-29

**Authors:** Lawrence P. Wackett

**Affiliations:** ^1^ Department of Biochemistry, Molecular Biology & BiophysicsBioTechnology InstituteUniversity of MinnesotaSt PaulMN55108USA

## Obafluorin and related antibiotics

### http://pubs.acs.org/doi/abs/10.1021/jo00092a025

Obafluorin is an antimicrobial β‐lactone compound produced by the bacterium *Pseudomonas fluorescens*. In this study, the compound was synthesized, analysed chemically and tested for biological activity.

## Triene β‐lactone antibiotics: Triedimycins

### https://www.ncbi.nlm.nih.gov/pubmed/1903377

The triedimycins are a class of spiro β‐lactones. They are not potent as antibiotics but show substantial *in vitro* antitumor activity against murine leukaemia cells.

## β‐Lactone natural products inactivate homoserine transacetylase

### http://www.nature.com/ja/journal/v64/n7/abs/ja201137a.html

The β‐lactone ebelactone A served as a lead compound to find potent inhibitors that inactivated homoserine transacetylase. Inhibition against that enzyme makes for a useful antimicrobial substance against pathogens, such as *Haemophilus influenzae*.

## Hymeglusin antifungal agent

### http://www.abcam.com/rr-hymeglusin-ab144274.html

This commercial website contains useful information on the β‐lactone antibiotic and antifungal agent, (R,R)‐hymeglusin.

## β‐Lactones as antibacterial agents: Patents

### https://www.google.com/patents/EP2254574A1?cl=en

This patent builds off a knowledge of β‐lactone natural products. Novel hydrophobic β‐lactone structures with alkenyl, alkynyl and phenyl groups were synthesized to test as novel antimicrobial substances.

## 4‐Methylene oxetanone

### http://webbook.nist.gov/cgi/cbook.cgi?ID=C674828&Mask=8

Methylene‐substituted β‐lactones are intermediates in the synthesis of mimics of β‐lactone natural products. The general characteristics of a simple methylene β‐lactone are available here.

## (‐)‐Lipstatin: Pubchem

### https://pubchem.ncbi.nlm.nih.gov/compound/71749817

Tetrahydrolipstatin is a β‐lactone antiobesity drug, currently available with or without prescription. The Pubchem website for this compound has information on chemical properties, biological assays and commercial availability.

## Fermentative production of lipstatin: Patent

### https://www.google.com/patents/EP2019869A1?cl=en

Lipstatin is the β‐lactone natural product produced by a *Streptomyces* species that can be reduced to make the drug tetrahydrolipstatin. The patent was filed to protect certain aspects of the fermentative production of the compound.

## β‐Lactones as synthetic intermediates for natural products

### http://oaktrust.library.tamu.edu/handle/1969.1/ETD-TAMU-2011-12-10494

The β‐lactone functional group is described here in this thesis for its value in the synthesis of natural products. Many of the natural products are not themselves β‐lactones, but the versatile reactivity of β‐lactones makes them useful as synthetic vehicles.

## (‐)‐Belactosin C: Pubchem

### https://pubchem.ncbi.nlm.nih.gov/compound/10666031#section=Top

This is the Pubchem page for the β‐lactone (‐)‐belactosin which has been tested for anticancer, antiviral and antimicrobial activity.

## Omuralide: Pubchem

### https://pubchem.ncbi.nlm.nih.gov/compound/Omuralide#section=Top

Omuralide has a β‐lactone ring fused to a second five‐membered ring. The compound has been shown to inhibit cysteine endopeptidases.

## Synthesis of the proteasome inhibitors salinosporamide A, omuralide and lactacystin

### http://www.organic-chemistry.org/Highlights/2005/28November.shtm

This page contains a general description of compounds containing β‐lactone and β‐lactam rings.

## Omuralide and vibralactone as different proteosome inhibitors

### http://onlinelibrary.wiley.com/doi/10.1002/anie.201308567/abstract

This study shows that minor structural differences can have a large impact on the biological targets of β‐lactone natural products.

## Proteosome mutant in complex with omuralide

### http://www.rcsb.org/pdb/explore.do?structureId=4r00

This link is to a Protein DataBank page. The depositors conducted a study on the binding of different drugs to a yeast mutant proteosome, and one of the compounds tested was the β‐lactone omuralide.

## Salinosporamide A: Wikipedia

### https://en.wikipedia.org/wiki/Salinosporamide_A

This natural product is produced by a marine bacterium. It contains a γ‐lactam‐β‐lactone bicyclic core structure. It is being used in early stage clinical trials to treat myeloma.

## β‐Lactone inhibitors of fatty acid synthase

### https://www.ncbi.nlm.nih.gov/pubmed/18710210

In this study, the hydrophobic β‐lactone natural products with alkyl chains were used as models for synthesizing 28 novel congeners to test for biological activity.

## β‐Lactone inhibitors of phospholipase a2: Patent

### https://www.google.com/patents/WO2016128131A1?cl=nl

This patent describes the synthesis of novel β‐lactones for the purpose of finding new inhibitors of phospholipase a2.

## Function‐oriented synthesis of β‐lactone proteosome inhibitors

### http://pubs.acs.org/doi/abs/10.1021/jm901098m

This study combined chemical synthesis and metabolic engineering to generate a series of salinosporamide analogues. Salinosporamide is natural product β‐lactone produced by a marine bacterium that acts a proteosome inhibitor.

## Ebelactone gene cluster

### https://www.researchgate.net/figure/242017149_fig2_Figure-6-Ebelactone-gene-cluster-of-S-aburaviensis-ATCC-31-860-Shown-are-the-putative

This site shows figures that include a gene cluster map depicting the genes encoding the biosynthesis of the β‐lactone natural product ebelactone, produced by a *Streptomyces* species.

## Obafluorin produced by *Pseudomonas fluorescens*

### https://www.jstage.jst.go.jp/article/antibiotics1968/37/7/37_7_802/_article

This is the original paper describing the discovery of the β‐lactone natural product obafluorin.

## Distribution of β‐lactam and β‐lactone producing bacteria

### https://www.ncbi.nlm.nih.gov/pubmed/7174535

This broadscale screening study, conducted in the pregenomic era, discovered a large number of β‐lactam and β‐lactone natural products produced by soil bacteria.

## β‐Lactones inhibiting bacterial virulence factors

### https://www.ncbi.nlm.nih.gov/pubmed/19206121

This study highlights the potential for β‐lactones to inhibit the central virulence regulator of bacterial pathogens, which could provide for an important new antibiotic mechanism.

## β‐Lactones for labelling active sites of bacterial enzymes

### http://onlinelibrary.wiley.com/store/10.1002/anie.200705768/asset/4600_ftp.pdf?v=1&t=iwlcf2zq&s=67ab989d840aa33b3af90bd8ffc311c07537d230

This communication illustrates the wide range of different bacterial enzymes inhibited by β‐lactone natural products.

